# The Effectiveness of Solution-Focused Brief Counseling on Marital Intimacy in Mothers of Children with Down Syndrome: A Randomized Clinical Trial: Solution-focused Counseling on Marital Intimacy in Mothers of Children with Down Syndrome

**DOI:** 10.31661/gmj.v12i.2747

**Published:** 2023-09-02

**Authors:** Mahshid Bokaie, Naeimeh Mirshafieian, Mir Saeid Jafari

**Affiliations:** ^1^ Research Center for Nursing and Midwifery Care, Non-communicable Diseases Research Institute, Department of Midwifery/Nursing, School of Nursing and Midwifery, Shahid Sadoughi University of Medical Sciences, Yazd, Iran; ^2^ International Campus of Shahid Sadoughi University of Medical Science, Yazd, Iran; ^3^ Department of Psychology, Islamic Azad University, Tehran South Branch, Tehran, Iran

**Keywords:** Marital Intimacy, Solution-focused Brief Therapy, Down Syndrome, Internet-based Intervention, Counseling

## Abstract

**Background:**

Parents of children with intelligence and motor problems, including Down syndrome have to spend more time babysitting resulting in less intimacy with their mates. Solution-focused brief therapy is one of the treatments presented in the field of marital intimacy. This study aimed to investigate the effectiveness of solution-focused counseling on marital intimacy in mothers of children with Down syndrome.

**Materials and Methods:**

In this randomized clinical trial study, 72 couples were selected among members of the Asemannili Society (Isfahan-Iran) from 19/01/2021 to 20/04/2021. The control group received an educational pamphlet for four sessions (without homework) every other week while the intervention group attended eight 90-minute online counseling programs once a week. Bagarozi Marital Intimacy Questionnaire was completed at baseline, after intervention (8th week), and follow-up period (12th week) by the women and their spouses.

**Results:**

The mean scores of marital intimacy between the two groups at baseline (online: 313.23 ± 70.86, pamphlet: 315.92 ± 41.45) compared to the 12th week (online: 370.13 ± 44.63, pamphlet: 332.42 ± 30.39) were significantly different. The analysis of the variance test with repeated observations showed that the effect of group, and time on the total score of marital intimacy and its other dimensions, were significant (P0.05) for women.

**Conclusion:**

Both online and pamphlet counseling can improve marital intimacy in mothers of children with Down syndrome, but online counseling appears to be more effective. Thus, this method is recommended for improving the marital intimacy of these women.

## Introduction

One of the basic human needs is the desire to establish intimate relationships and strive for belonging. To develop intimacy, marriage offers a unique opportunity that goes beyond intimate relationships with friends and relatives [1]. Dissatisfaction with intimacy may increase disagreements, reduces marital satisfaction, and cause emotional-psychological problems [2]. To address the challenges in marital relationships, establishing an intimate relationship, transferring feelings- thoughts, and talking about one’s needs have been proposed. Such mutual communication and cooperation require personal growth [3]. A large number of couples who refer to counseling and psychotherapy center have failed to obtain a satisfactory level of intimacy [4].

The solution-based treatment is based on solution-making strategies, not problem-solving skills. The underlying assumption of solution-based therapy indicates that investigating the basic problem is not needed in leading the counseling discussion because the cause of each problem is not necessarily related to its solution. In other terms, this therapy presupposes that all individuals are equipped with the necessary resources to make a change [5]. This approach leads clients to create the desired future vision in their daily lives via cooperation in outlining prospects based on their past successes, strengths, and resources [6]. Multiple evidence confirms that parents of children with intelligence problems are more probable to encounter social, economic, and emotional problems that are often limited, destructive, and pervasive [7]. In such a situation, although all family members are harmed and their functions are disturbed [8], mothers are the most vulnerable group due to their traditional role as caregivers. As a result, mothers of children with intellectual problems face numerous psychological and mental health challenges, including problems caused by taking care of children with specific needs [9]. Compared to mothers of children with no medical problems, mothers of children with mental disabilities have higher levels of anxiety [10] and more feelings of shame and embarrassment, but lower levels of general health and psychological well-being [11].

A study conducted in Iran found that the solution-focused counseling approach increased marital intimacy in the intervention compared to the control group [12]. Another research concluded that the solution-based intervention was effective in enhancing the resilience of mothers of mentally retarded children in Arak City, Iran [13]. Based on the results of studies conducted in Iran, group, sexual counseling sessions can improve sexual satisfaction among Iranian women [14][15]. Considering the current Coronavirus pandemic, this study was conducted through online platforms. Given the scarcity of studies on parents of children with Down syndrome, this study aimed to investigate the effect of solution-focused online counseling on marital intimacy in mothers with Down syndrome children.

## Materials and Methods

**Table:** Table1. Comparison of the Demographic Characteristics of the Two Groups

**Variables**	**Online group** **(n=31)**		**pamphlet group** **(n=36)**				
	**Mean**	**SD**	**Mean**	**SD**	**t**	**df**	*P
Women's age (years)	38.47	7.05	40.64	9.76	1.02	64	0.31
Age of spouses (years)	42.17	6.68	43.86	8.86	0.36	64	0.39
Length of marriage (years)	14.32	6.46	17.47	8.57	1.22	65	0.10
Number of pregnancies	2.10	0.70	2.08	0.73	0.27	65	0.94
Number of births	1.84	0.58	1.78	0.59	0.63	65	0.67
Age of A child with Down syndrome (years)	9.11	5.95	9.75	7.70	0.07	62	0.72
Age of the woman at the time of conception of the child with Down syndrome (years)	28.55	5.75	29.81	6.60	0.59	65	0.41

**SD:** Standard deviation; ^*^: Independent t-test

### Subjects and Randomization

This randomized clinical trial was conducted at the Asemannili Society of Isfahan/IRAN, which is a center for patients with Down syndrome (From January to April 2021). This study was approved by the Ethics Committee of Shahid Sadoughi University of Medical Sciences (approval code: IR.SSU.REC.1399.152) and registered in the Iranian Clinical Trial Registration System (code: IRCT20200620047846N1). Also, all participants signed the informed consent before the study. The intervention group received eight online sessions of marital intimacy counseling (90 minutes per week) conducted based on the solution-focused approach. The control group was provided with some educational pamphlets in four sessions every week. The educational contents covered through the intervention period were designed based on a review of the literature and opinions of the research team (supplement 1). Blinding was not possible due to the specific type of intervention method.

### Sample Size Calculation

Convenient sampling was utilized to conduct this study, and participants were randomly assigned to both intervention and control groups. The sample size was initially calculated as 36 in each group (Formula 1), which increased to 36 after considering the 10% probable dropouts.

Formula1.


n = \frac{(z1 + z2)^2 (2s^2)}{d^2}

Where, z1 for 95% confidence interval=1.96, Z2 for 80% test power=0.84, s=the mean standard deviation of intimacy scores in the two groups, d=the minimum difference between mean scores of intimacies between the two groups, which showed a significant difference and was considered as P<0.05.

### Participants

The study population included 250 couples who were members of the Isfahan Asemannili Society. Of these couples, 118 did not meet the inclusion criteria and 60 were unwilling to participate in the study. So, the remaining couples (n=72) were asked to enter the study after completing the online informed consent form. The study participants included 72 mothers of children with Down syndrome who were randomly assigned to the intervention and control groups. The intervention group received online counseling based on the solution-focused approach (n=36) and the control group members obtained related pamphlets (n=36). Randomization was performed via the website of random allocation http://www.randomization.com.

### Inclusion Criteria

Participants with the following criteria entered the study: willing to participate in the study, having a smartphone, being Iranian, being a resident in Isfahan, having the ability to read and write, being married, being the only partner, having a child ≥2 years of age with Down syndrome, and being a member of Isfahan Asemannili Society.

### Exclusion Criteria

Alcohol or drug consumption, taking medicines affecting sexual function, such as psychiatric drugs, suffering from diseases affecting sexual function, such as diabetes, applicants for other support such as psychological services or participating in other counseling programs.

### Data Collection

Demographic data and the Marital Intimacy Questionnaire were collected through an electronic link in the online and pamphlet groups. Questions about demographic characteristics include age, employment status, level of education, history of pregnancy, number of births, how many years have passed since their marriage, the age of the child with Down syndrome, the birth rank in the family, the age of the woman at the time of this child’s pregnancy, marriage with first degree relatives. Also, the Marital Intimacy Needs Questionnaire (MINQ) was used for the assessment of the marital intimacy of spouses at baseline, end of 8th and 12th weeks.

### Bagarozzi Marital Intimacy Questionnaire

This 44-item questionnaire, designed by Bagarozzi (2001) [16], measures nine dimensions of marital intimacy, including emotional, intellectual, physical, social and recreational, aesthetic, sexual, spiritual, psychological, and temporal intimacy. The questions should be answered on a 10-point Lickert scale. All subscales, except for the subscale of spiritual intimacy, have five questions that should be answered on a 5-point scale ranging from 1 (this need does not exist in me at all) to 10 (this need is strong in me). The minimum and maximum attainable total scores in these subscales are 5 and 50, respectively. The spiritual intimacy includes six questions and its attainable scores are within the range of 1-10. The minimum and maximum attainable scores of this subscale are 6 and 60, respectively. Finally, the subscale of intimacy in spending time is scored qualitatively, in such a way that all three questions of this subscale are calculated based on the average answers of people to other subscales. The maximum attainable score in this questionnaire is 440. The Cronbach alpha coefficient was calculated as 0.95 for the original version and 0.93 for the Persian version which was assessed by Etamadi (1385) [17][18].

### Intervention

Participants were randomly assigned to two groups. The intervention group received eight solution-focused counseling sessions online and the control group received pamphlets with the same content. The online counseling sessions were conducted by a master’s student of midwifery counseling, who had acquired the necessary skills in this field. Counseling took place under the supervision of a supervisor and a counselor who specializes in solution-focused and couple therapy [19][20]. A reminder SMS was sent to the participants before each session. At first, both groups were created on WhatsApp titled "Online Counseling Group" and "Pamphlets Group", and then the WhatsApp phone number of each person was received to add them to the groups. The participants of the online counseling group were provided with one-month free internet packages. The online counseling session was held in Skyroom virtual spaces and groups. At the beginning of the meeting, the subject of the project and its goals were presented to the two groups of participants.

Following the method of the study, the control group received the intervention in the form of Word formatted pamphlets during four sessions, every other week. Unlike the test group, the control group members were not supposed to do any homework and send it to the WhatsApp group. Eight 90-minute sessions were held one day a week for the participants of the intervention group. At each stage of the research, the subjects’ permission to leave the project was explained. In each session, in addition to a review of the assignments given to the participants in the previous session, they were asked about the effectiveness of the counseling and their progress. They were also asked to talk about the changes they experienced in their marital intimacy based on the content of the sessions. The interventions lasted from 2021-01-19 to 2021-04-20.

### Data Analysis

Data extracted from the questionnaire were analyzed using SPSS software version 22 (Statistical Package for the Social Sciences, version 22, SPSS Inc, Chicago, Illinois, USA). The descriptive-inferential statistics were employed to analyze the data. The collected data are displayed in Table-3 and -4. Inferential statistics consisted of independent t-test (quantitative variables, including age, number of delivery, and so forth.), Chi-square (qualitative-nominal variables, including husband’s job, and so forth.), and Mann-Whitney (qualitative rank-order variables, including education level, and so forth.). The repetitive measures analysis of variance was also administered. Descriptive statistics, including frequency, percentage, mean, and standard deviation of qualitative variables were applied to present and describe the information, prepare tables, and calculate the percentage, mean, and standard deviation of the data, while inferential statistics were performed to analyze the differences in mean scores. The significance level (P<0.05) was considered.

## Results

**Figure-1:**
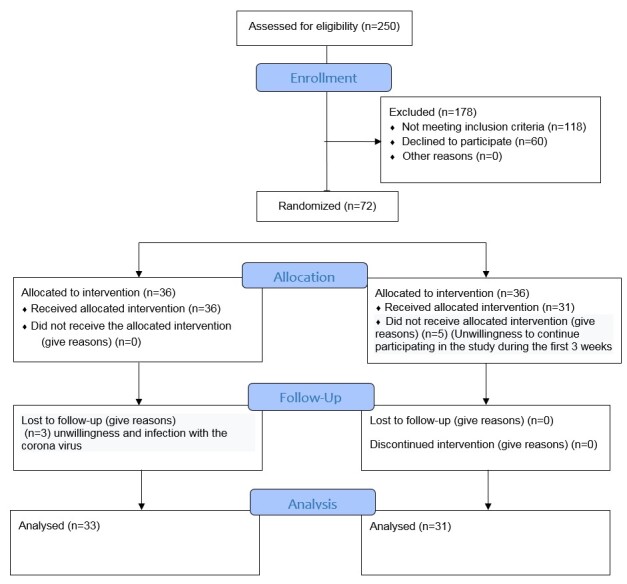
Figure-1 .CONSORT flowchart

**Table:** Table2. Comparing the Frequency Distribution of Women’s Jobs, Spouses’ Jobs and Marriage with 1st Degree Relatives between the Two Groups

Variables		Online group 31 couple		pamphlet group 36 couple				
		Number	Percentage	Number	Percentage	χ ^2^	df	P*
Occupational status(women’s)	Housewife	27	87.1	32	88.9	-	-	0.82
	Employed	4#	12.9	4#	11.1			
Spouse's job	Worker	8	25.8	10	27.8	0.37	3	0.95
	Employee	12	38.7	12	33.3			
	Self-employment	10	32.3	12	33.3			
	Unemployed	1#	3.2	2#	5.6			
Marriage with relatives	No	28	90.3	31	86.1	-	-	0.59
	Yes	3#	9.7	5	13.9			

^*^: Chi-Square test;**#:**Fisher's exact test

Totally 250 eligible women for the study were assessed and finally, 72 of them were enrolled. One person from the online counseling group dropped out in the first week of the intervention due to her unwillingness to continue participating in the study, and one person dropped out in the second week because she did not have time to attend the sessions (employed). And three others dropped out from the study in the third week, one due to coronavirus infection and being in quarantine, and the other two due to the spouse’s unwillingness and uncooperativeness for completion of the homework, and final analyses were performed on 31 couples in online intervention and 36 couples who received pamphlet (see Figure-1: CONSORT flowchart).

The mean age of women was 38.47 ± 7.05 years in the online counseling group and 40.64 ± 9.76 in the pamphlet-receiving groups, respectively. Meanwhile, the mean age of the participants’ spouses was 42.17 ± 6.68 years in the online counseling group and 43.86 ± 8.86 years in the control group who received the educational pamphlets, respectively. According to the independent t-test, the mean age of women, the mean age of their spouses, the duration of the marriage, the number of pregnancies, the number of deliveries, the age of a child with Down syndrome, and the age of the mother at the time of pregnancy had no significant difference between the two groups (P>0.05, Table-1).

**Figure-2:**
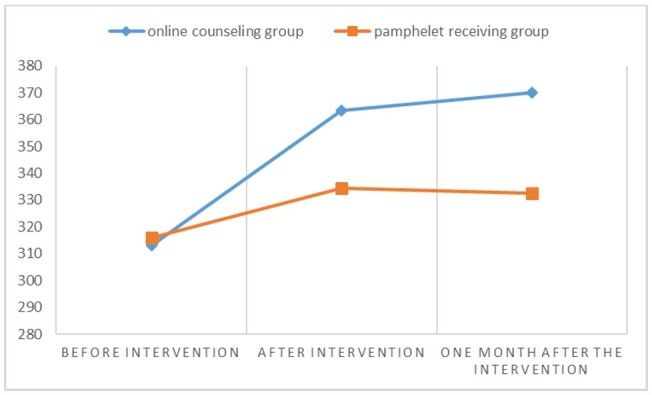
Figure 2. The average score of total marital intimacy of women at different times in the two groups

**Figure-3:**
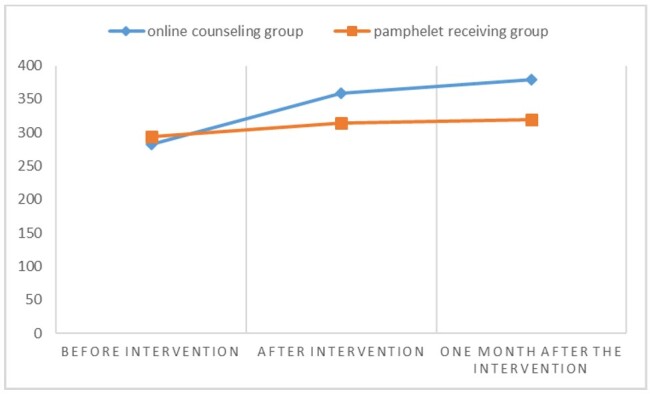
Figure 3. The mean score of the total marital intimacy of spouses at different times in the two groups

Comparing the frequency distribution of women’s jobs, spouses’ jobs, and marriage with 1st degree relatives between the two groups is presented in Table-2. Most women and their husbands in both groups had academic education. The majority of children with Down syndrome in both groups were the first child of their families. The Mann-Whitney test showed no significant difference between the two groups regarding the parents’ level of education and birth order of a child with Down syndrome (P>0.05, Table-3). According to the repeated measures analysis of variance, the passage of time had a significant impact on marital intimacy scores of women in sexual, spiritual, social and recreational, and temporal domains (P<0.001) but group membership did not have a significant effect on these domains (P>0.05). So, the mean scores of marital intimacy increased over time in the domains of sexual, spiritual, social and recreational, and temporal but the difference between the two groups was not significant. In general, group membership and time had significant impacts on the total score of marital intimacy and its domains. The mean score of overall marital intimacy and its domains, except for the social and recreational dimension, increased over time, but this increase was significantly higher in the online counseling group than in the pamphlet-receiving group. Based on the repeated measures analysis of variance, time and group membership had a significant impact on the total score of marital intimacy and all its domains (P<0.05). The mean score of marital intimacy and all its domains increased over time and this increase was significantly higher in the online counseling group compared to the pamphlet-receiving group (Figure-2). Based on the repeated measures analysis of variance, time and group membership had a significant impact on the total score of marital intimacy and all its domains (P<0.05) in spouses. The mean score of marital intimacy and all its domains increased over time and this increase was significantly higher in the online counseling group than in the pamphlet-receiving group in their spouses (Table-4). Based on the repeated measures analysis of variance, time and group membership had a significant impact on the total score of marital intimacy and all its domains (P<0.05). The mean score of marital intimacy and all its domains increased over time and this increase was significantly higher in the online counseling group compared to the pamphlet-receiving group in their spouses Table-5 and Figure-3.

## Discussion

**Table:** Table3. Comparison of Frequency Distribution of Women’s Education Level, Spouse’s Education Level and the Birth Rate of the Child with Down Syndrome between the Two Groups

Variables		Online group 31 couple		Pamphlet group 36 couple			
		Number	Percentage	Number	Percentage	Z	P*
Education level of women	Under diploma	3	9.7	4	11.1	0.33	0.73
	Diploma	7	22.6	9	25.0		
	Above diploma	21	67.7	23	63.9		
Education level of spouses	Under diploma	7	22.6	13	36.1	1.87	0.06
	Diploma	6	19.4	11	30.6		
	Above diploma	18	58.1	12	33.3		
The birth rank of the child with Down syndrome	First	20	64.5	22	61.1	0.34	0.73
	Second	10	32.3	12	33.3		
	Third	1	3.2	2	5.6		

^*^: Mann-Whitney test

**Table:** Table4. Mean Score of Total Marital Intimacy and its Domains at Different Times in Two Groups (Women)

Dimensions of marital intimacy	Time	Online group		Pamphlet group		*P-value	*P-value
		(N=31)		(N=36)		(Time effect)	(Group effect)
		Mean	SD	Mean	SD		
Emotional	Base Line	35.65	7.90	35.11	4.96		
	After Intervention	42.00	5.39	37.61	3.59	0.001>	.0080
	Follow-up	43.00	4.96	38.11	3.65		
Psychological	Base Line	35.64	8.65	35.53	5.31		
	After Intervention	41.13	5.50	37.39	4.07	0.001>	.0400
	Follow-up	42.06	5.25	37.72	4.12		
Rational	Base Line	58/35	46/8	86/35	10/5		
	After Intervention	41.39	5.48	37.17	4.29	0.001>	0.030
	Follow-up	42.03	5.04	37.39	4.19		
Sexual	Base Line	35.68	9.24	36.94	5.85		
	After Intervention	41.39	6.53	38.31	4.59	0.001>	0.239
	Follow-up	42.00	6.29	38.58	4.40		
Physical	Base Line	36.52	8.33	36.56	4.75		
	After Intervention	42.06	5.38	38.03	3.95	0.001>	0.023
	Follow-up	42.74	5.25	38.06	3.91		
Spiritual	Base Line	42.81	11.49	43.22	8.17		
	After Intervention	49.42	7.90	45.03	6.25	0.001>	0.112
	Follow-up	50.39	7.25	45.33	6.03		
Aesthetic	Base Line	34.61	10.11	33.83	6.06		
	After Intervention	40.03	6.91	35.11	5.25	0.001>	.0220
	Follow-up	40.71	6.39	35.25	5.16		
Recreational and social	Base Line	33.71	10.46	34.89	7.60		
	After Intervention	39.84	7.27	41.00	5.68	0.001>	0.836
	Follow-up	40.71	7.04	37.31	5.19		
Temporal	Base Line	23.00	5.35	23.97	2.83		
	After Intervention	26.00	3.28	24.67	2.40	0.001>	.330
	Follow-up	26.48	3.19	24.67	2.40		
Total score of marital intimacy	Base Line	313.23	70.86	315.92	41.45		
	After Intervention	363.32	47.05	334.31	31.38	0.001>	.0470
	Follow-up	370.13	44.63	332.42	30.39		

**SD:** Standard deviation;^*^: Repeated Measures

**Table:** Table5.Mean Score of Total Marital Intimacy and its Domains at Different Times in Two Groups (Spouses )

Dimensions of marital intimacy	Time	Online group (N=31)		Pamphlet group (N=36)		*P-value	*P-value
						(Time effect)	(Group effect)
		Mean	SD	Mean	SD		
Emotional	Base Line	31.42	6.83	32.51	4.68		
	After Intervention	41.03	5.69	35.52	3.82	0.001>	0.002
	Follow-up	43.42	4.54	36.85	3.54		
Psychological	Base Line	32.26	7.72	33.03	4.36		
	After Intervention	40.81	5.90	35.30	3.90	0.001>	0.001>
	Follow-up	43.03	4.55	35.67	3.76		
Rational	Base Line	32.68	7.36	34.03	4.25		
	After Intervention	41.19	5.81	09/36	4.01	0.001>	0.006>
	Follow-up	42.81	4.42	36.48	3.90		
Sexual	Base Line	32.35	8.09	34.18	4.79		
	After Intervention	41.03	6.38	36.18	3.75	0.001>	0.016
	Follow-up	43.39	5.38	36.88	3.65		
Physical	Base Line	32.74	7.67	34.00	4.09		
	After Intervention	41.45	5.69	36.24	3.82	0.001>	0.002
	Follow-up	43.90	4.53	36.94	3.64		
Spiritual	Base Line	38.74	9.94	40.42	7.07		
	After Intervention	48.84	7.51	43.33	5.44	0.001>	0.023
	Follow-up	51.42	5.69	43.82	5.45		
Aesthetic	Base Line	31.48	8.63	32.45	4.99		
	After Intervention	39.84	7.14	33.91	4.30	0.001>	0.004
	Follow-up	41.90	5.49	34.30	4.38		
Recreational and social	Base Line	30.87	9.01	31.85	6.09		
	After Intervention	39.55	7.77	34.61	4.59	.0010>	0.020
Follow-up	42.10	6.06	35.21	4.19		
Temporal	Base Line	20.52	4.80	21.91	2.49		
	After Intervention	25.55	3.66	23.18	2.40	0.001>	.0350
	Follow-up	27-Mar	2.71	23.39	2.34		
Total score of marital intimacy	Base Line	06/283	62.20	294.45	34.95		
	After Intervention	359.32	50.51	314.36	29.00	0.001>	0.002
	Follow-up	379	37.25	319.55	28.40		

**SD:** Standard deviation;^*^: Repeated Measures

The present study aimed to examine the effect of solution-focused counseling on marital intimacy in mothers of children with Down syndrome. Based on the findings, the total score of marital intimacy and its subscales increased significantly after eight and 12 weeks from the study compared to the prestudy. This finding was supported by Hosseini’s study [21]. The results of the mentioned study indicated that the solution approach focused on increasing the overall marital intimacy of the participating members in the dimensions of emotional, psychological, and sexual intimacy was spending time and in other dimensions of marital intimacy including intellectual, physical, spiritual, aesthetic and social-recreational no significant difference was observed between the two groups [12]. Our study was not consistent with Hosseini’s study in the variations in the intervention’s type, duration, method, and contents.

Kamali et al. (2019) also noted the effective factors in increasing marital intimacy included family, time spent together/length of marital relationship, dedication and mutual forgiveness, gratitude, new shared activity, parenting, shared social networks, and religion [7]. In this vein, Ahmadi Khoei et al. reported that divorce prevention training increased the intimacy and quality of communication between couples [8]. Nazari et al. (2019) found that training communication skills via enriching couples’ relationships through the Olson approach could be effective in promoting marital intimacy [9] since communication skills have been confirmed as one of the predictors of marital intimacy explaining and predicting 46% of marital intimacy [10]. In explaining these cases, it can be said that sexual health counseling and training communication skills improve the marital intimacy of couples and encourage them to express their thoughts and feelings in the field of couple relationships. All recent studies were consistent with our study, although the studied couples were different.

Burke et al. (2008) noted that since mothers of children with Down syndrome were at lower levels of mental health, they may need more support and health services to improve their behavioral-management skills, which in turn improves the family’s mental well-being. Policy-makers and authorities are recommended to employ these findings to hold educational courses for caregivers of people with intellectual disabilities and identify specific strategies to improve the child’s behavior and mother’s management skills and mental well-being [22]. In our study, women of children with Down syndrome wanted to continue sexual counseling. One of the most highlighted findings of this study was significantly higher marital intimacy and total scores in all domains at 8 and 12 weeks after the study compared to baseline scores. A possible reason for this finding may be attributed to the fact that women participating in a solution-based approach training program can influence the domain of intimacy in men. Based on the literature, no studies have yet investigated the impact of this approach on marital intimacy in men. The solution-focused approach was found to enhance the vitality and resilience of physically disabled students [23] as well as the happiness and emotion regulation of couples [24]. Solution-focused brief therapy reduced depression, increased marital satisfaction in married women [25], and could enhance marital satisfaction in mothers of students with intellectual disabilities [26]. Yousefi et al. (2018) compared the effectiveness of acceptance-commitment and solution-focused group counseling approaches on the performance of couples on the verge of divorce. Zakhirehdari et al. showed the effectiveness of cognitive-behavioral couple therapy in improving marriage performance and marital intimacy of couples. Tavaloli et al. showed the effectiveness of marriage enrichment training of TIME plan on improving marital intimacy and psychological security of women [27][28][29]. These studies showed that counseling approaches can be associated with improving the performance and marital intimacy of women in some of the studied couples. As they mentioned, all approaches of group counseling were significantly effective in marital satisfaction. In the present study, the solution-focused brief approach was more effective in increasing marital intimacy in mothers of children with Down syndrome. In the post-test to follow-up phases, time did not affect on reducing the effectiveness of this treatment. Our findings showed that solution-focused training increased marital intimacy in the intervention group compared to the control group. These results were in line with some studies investigating the effectiveness of solution-focused brief couple therapy on couples’ happiness and emotion regulation [24].

The solution-focused brief therapy is a useful approach [30][31][32], the solution-focused brief therapy reduced marital stress among different populations and in a variety of settings, including couple therapy, family therapy, treatment of patients with intellectual deficits, treatment of sexual abuse, and major depressive disorder [33][34][35][36][37]. Couples who were influenced by short-term family training had significant progress in their marital adaptation and satisfaction [38]. Similarly, solution-focused group couple therapy increased marital consensus and satisfaction of couples [39]. In congruence with these findings, an investigation of the efficiency of solution-focused group therapy for couples indicated a significant improvement in the marital satisfaction level at the end of the intervention [21][40]. This treatment method had an enhancing effect on marital satisfaction, marital adjustment, quality of marital relationships, intimacy, and affection expression, which reduced the rates of divorce and resolved many marital conflicts.

To shed more light on these findings, one may notice that the solution-based approach emphasizes the present and future instead of drowning clients in the past and rooting out the causes of the problem [41]. The solution-focused brief approach is interested in family change but does not take into account why the problems emerge in the family and is mainly focused on the solutions. While therapists and their clients gradually talk more about the solutions, they develop a belief in the truth and reality of what they are talking about. This treatment has different components, including developing positive viewpoints, avoiding labeling, and believing in the ability of clients. Our findings indicated that solution-focused brief counseling significantly increased marital intimacy and all its dimensions compared to the pre-intervention status. Furthermore, the online counseling group outperformed the pamphlet-receiving group significantly. Another noteworthy point was that counseling in women was indirectly associated with improvement in their partner’s sexual intimacy.

## Conclusion

Although solution-focused brief counseling, whether in the form of online education or pamphlets, could improve marital intimacy and its (emotional, psychological, intellectual, sexual, physical, spiritual, aesthetic, social-recreational, and temporal intimacy dimensions) in mothers of children with Down syndrome, the effect of online counseling was significantly higher than that of receiving pamphlet. This finding can be justified by referring to specific characteristics of online counseling, including employing electronic facilities, representing content in the form of video chat, and providing the possibility of reviewing the educational content as frequently as required by the participants and their spouses. Consequently, members of the online counseling groups could learn the components of intimacy, acquire communication skills to associate among various components of intimacy and develop a realistic view of Down syndrome by correcting inefficient beliefs about marital intimacy after encountering a child with Down syndrome. In this vein, participants of the present study learned to use the methods of establishing intimate marital relationships during the COVID-19 pandemic efficiently for improving their marital intimacy. We offer couples therapy counseling for these couples.

## Conflict of Interest

No potential conflict of interest has been reported by the authors.
